# Oligometastasis in breast cancer—current status and treatment options from a radiation oncology perspective

**DOI:** 10.1007/s00066-022-01938-x

**Published:** 2022-05-08

**Authors:** Marc D. Piroth, David Krug, Petra Feyer, René Baumann, Stephanie Combs, Marciana-Nona Duma, Jürgen Dunst, Gerd Fastner, Rainer Fietkau, Matthias Guckenberger, Wulf Haase, Wolfgang Harms, Thomas Hehr, Felix Sedlmayer, Rainer Souchon, V. Strnad, Wilfried Budach

**Affiliations:** 1 Dpt. of Radiation Oncology, Helios University Hospital Wuppertal, Witten/Herdecke University, Heusnerstr. 40, 42283 Wuppertal, Germany; 2 University Hospital Schleswig-Holstein, Kiel, Germany; 3 formerly Vivantes Hospital Neukoelln, Berlin, Germany; 4 St. Marien Hospital Siegen, Siegen, Germany; 5 University Hospital Klinikum rechts der Isar, Technical University of Munich (TUM), Munich, Germany; 6 University Hospital Jena, Jena, Germany; 7 University Hospital Salzburg, Paracelsus Medical University, Landeskrankenhaus, Salzburg, Austria; 8 University Hospital Erlangen, Erlangen, Germany; 9 University Hospital Zurich, University of Zurich, Zurich, Switzerland; 10 formerly St.-Vincentius-Hospital Karlsruhe, Karlsruhe, Germany; 11 St. Claraspital Basel, Basel, Switzerland; 12 Marienhospital Stuttgart, Stuttgart, Germany; 13 formerly University Hospital Tübingen, Tübingen, Germany; 14 University Hospital Düsseldorf, Düsseldorf, Germany

**Keywords:** Breast cancer, Oligometastases, Radiotherapy, Stereotactic body radiotherapy, OMD

## Abstract

Evidence from a few small randomized trials and retrospective cohorts mostly including various tumor entities indicates a prolongation of disease free survival (DFS) and overall survival (OS) from local ablative therapies in oligometastatic disease (OMD). However, it is still unclear which patients benefit most from this approach. We give an overview of the several aspects of stereotactic body radiotherapy (SBRT) in extracranial OMD in breast cancer from a radiation oncology perspective. A PubMed search referring to this was conducted. An attempt was made to relate the therapeutic efficacy of SBRT to various prognostic factors. Data from approximately 500 breast cancer patients treated with SBRT for OMD in mostly in small cohort studies have been published, consistently indicating high local tumor control rates and favorable progression-free (PFS) and overall survival (OS). Predictors for a good prognosis after SBRT are favorable biological subtype (hormone receptor positive, HER2 negative), solitary metastasis, bone-only metastasis, and long metastasis-free interval. However, definitive proof that SBRT in OMD breast cancer prolongs DFS or OS is lacking, since, with the exception of one small randomized trial (*n* = 22 in the SBRT arm), none of the cohort studies had an adequate control group. Further studies are needed to prove the benefit of SBRT in OMD breast cancer and to define adequate selection criteria. Currently, the use of local ablative SBRT should always be discussed in a multidisciplinary tumor board.

## Introduction

Traditionally, treatment for metastatic breast cancer has been regarded as palliative, with the corresponding therapeutic implications. However, this view has been challenged in recent years due to observations in other solid tumors. In 1968, Philip Rubin proclaimed that there are considerable prognostic differences in metastatic disease, and he asked: “Are metastases curable?” [1]. In the 1990s, the radiation oncologists Samuel Hellman and Ralph. R. Weichselbaum published their hypothesis of oligometastatic disease (OMD), which is to be understood as a transitional state between locally confined disease and widespread metastatic disease [2]. It was postulated that a subset of patients with a small number of macroscopic metastases may have a limited microscopic tumor burden and could be treated with “curative” local therapy and “adjuvant” drug therapy.

This approach is supported by the success of surgical series. In cohorts including several histological tumor sites with mostly colorectal cancer or sarcoma patients, surgical removal of liver or lung metastases resulted in 5‑year survival rates of up to 50 % depending on the risk constellation [3–7]. In the past, this hypothesis was further supported by several single-arm studies on stereotactic body radiotherapy (SBRT). However, most of these studies showing the efficacy of local ablative therapy with long-term survival in a subset of patients with oligometastatic disease enrolled a very limited number of breast cancer patients [8]. In recent years, further data have indicated that cure might be potentially possible in prognostically favorable cases of OMD [9–11]. However, although significant prognostic differences and various treatment approaches in the metastatic disease group are known, a definition of a favorable OMD group with the potential for cure is unclear.

In 2020, a group of 20 international experts of the ESTRO and EORTC OligoCare project developed a comprehensive system for characterization and classification [12], which aims to take into account the different clinical scenarios of OMD. Criteria for subclassification include timing of OMD (synchronous vs. metachronous), history of previous OMD (repeat vs. de novo OMD), systemic therapy at the time of OMD diagnosis (oligorecurrence vs. oligoprogression), response to systemic therapy (oligopersistence vs. oligoprogression), and previous history of polymetastatic disease (induced OMD). This classification was recently validated in a retrospective single-center cohort of 385 patients with OMD [13].

This classification is very useful for further studies but can only in part be applied to the currently available data. In current clinical trials, a maximum of 3–5 metastases are accepted in the context of OMD. However, whether the definition of OMD should strictly rely on a maximum number of metastatic sites is a matter of debate. Probably, the criteria used so far to classify OMD versus no OMD do not do sufficient justice to the biology of OMD. There is much to be said for a spectrum of diseases, with a fluent transition between local and systemic disease [14], and depending on this, a more or less pronounced benefit of local therapy.

An ESTRO-ASTRO OMD consensus document states on the one hand that “the possibility to safely deliver curative intent metastasis-directed radiotherapy determines the maximum number” [15], but on the other hand states that the ability to safely treat all oligometastases with radiotherapy does not mean that one should treat every patient irrespective of other prognostic factors.

In this review, we try to highlight the current data on oligometastatic breast cancer with regard to stereotactic body radiotherapy (SBRT) for extracranial disease sites. In addition, several aspects such as prognostic factors and systemic therapy will be addressed.

## Methods

A PubMed search was conducted to extract relevant articles from 2000 to 2021. A search was performed using the following terms: (oligometastases OR oligometastasis OR oligometastatic disease OR oligo metastatic disease) AND (breast cancer OR breast neoplasm OR breast) AND (radiotherapy OR ablative radiotherapy OR stereotactic radiotherapy OR radiation).

We give an overview of results of prospective and retrospective studies using SBRT to treat extracranial disease sites for patients with oligometastatic breast cancer, as well as an overview of prognostic factors and further treatment aspects.

### Summary of trials of SBRT for patients with oligometastatic breast cancer

In Table 1, relevant studies including breast cancer patients with OMD treated with SBRT are presented. Overall, the 10 studies included 505 patients. In six studies, the design was retrospective and in four studies prospective. All but one study were single-arm studies without a control arm. Regarding the only randomized study by Tsai et al., which reported a preplanned subgroup analysis on patients with oligoprogressive breast cancer, only an interim analysis in abstract form is available [16].

**Table 1:** Summary of trials on radiotherapy for oligometastases in breast cancer

First author, year	*N*	FU (months)	Study design	Number of metastases	Subtype of OMD	Sites	Previous systemic therapy	Fractions/doses	Toxicity	**–**	OS	PFS	LC
Tsai et al., 2021 [15]	44	–	Prospective, randomized	1: 25 % > 2: 75 %	Oligoprogression	**–**	100 %	SBRT (no further information available)	≥ Grade 2: 26.7 %	**–**	**–**	4.5 months (SBRT) vs. 4.3 months	**–**
David et al., 2020 [16]	15 (19 met)	24	Prospective, single arm	1: 73 % 2: 27 %	Synchronous OMD 13 % Metachronous OMD 87 %	Bone 100 %	87 % (endocrine)	1 fx/20 Gy	Grade 1: 67 % Grade 2: 27 % No grade ≥ 3	2‑year	100 %	65 %	100 %
Milano et al., 2019 [10]	48 (102 met)	52.8	Prospective, single arm	1: 39.6 % 2: 31.3 % 3: 14.6 % 4: 6.2 % 5: 8.3 %	Synchronous OMD (17 %) Metachronous OMD (83 %)	Bone 25 % Lung, lymph nodes, liver, adrenal 75 %	91 % (56.2 % endocrine)	5–10 fx (10 fx in 56.3 %; 1 patient < 5 fx)/ 3–17 Gy per fraction (in all but 2 patients)	n/a	5‑year 10-year	5‑year: 31 % (excluding bone) vs. 83 % bone only 10-year: 31 % (excluding bone) vs. 83 % bone only	n/a	10-year: 73 % (excluding bone) vs. 100 % bone only
Trovo et al., 2018 [19]	54 (92 met)	30	Prospective, single arm	1: 50 % 2: 35.1 % 3: 11.1 % 4: 1.9 % 5: 1.9 %	Synchronous OMD 74 % Induced OMD 26 %	Bone 65.2 % Lymph nodes 25 % Lung/liver 9.8 %	89 % (17 % endocrine)	3 fx/30–45 Gy (*n* = 44) or 25 fx/60 Gy (*n* = 10)	No grade ≥ 3	1‑year 2‑year	97 % 95 %	75 % 53 %	97 % 95 %
Scorsetti et al., 2016 [17]	33 (47 met)	24	Observational, single arm	1: 63.6 % 2: 30.3 % 3: 6.1 %	n/a	Liver, lung 100 %	90.9 %	3 fx/56.25–75 Gy (for liver met); 4 fx/48 Gy (*n* = 13) or 3 fx/60 Gy (lung met)	Grade 3: 18 % No grade ≥ 3	Median 1‑year 2‑year	48 months 93 % 66 %	11 months 48 % 27 %	n/a 98 % 90 %
Lemoine P et al., 2021 [18]	44	40.8	Retrospective, single arm	1–5	n/a	Bone 44.4 % Liver 40.7 % Lung 11.1 %	52.8 % (29.5 % endocrine)	3–10 fx (median 3)/15–54 Gy (median 40)	Grade 1: 25 %, grade 2: 7 %, no grade ≥ 3	Median 1‑year 2‑year 3‑year	n/a 93 % 87 % 81 %	31.2 months 81 % 58 % 45 %	n/a 100 % 100 %
Tan et al., 2021 [21]	120 (193 met)	15.3	Retrospective, single arm	1–5	OMD 55 % Oligoprogression 30 % OMD with intention of local control of dominant tumor 15 %	Bone 59.20 % Liver 20 % Lung 17.5 %	91.7 % (84 % endocrine)	4–5 fx/48–52 Gy 3–5 fx/30–60 Gy 5 fx/30–40 Gy 2 fx/24 Gy 4–5 fx/30–35 Gy (regimen depending on organ affected)	Grade 3: 4.2 % (3 radiation pneumonitis, 2 vertebral fractures)	Median 1‑year 2‑year	53.16 months 83.5 % 70 %	11 months 45 % 32 %	n/a 89 % 86.6 %
Wijtunga et al., 2021 [31]	79 (103 met)	50	Retrospective, single arm	1: 80 % > 1: 20 %	Metachronous OMD (44 %) Oligoprogression 47 % Oligopersistence 9 %	Bone 93 % Lymph nodes 4 % Lung 2 % Skin 1 %	n/a	1 fx/18–24 Gy 3 fx/24–30 Gy 4 fx /40–48 Gy 5 fx/25–35 Gy 8 fx/40 Gy	No grading given Toxicity/symptoms: At baseline: 66 % Acute (up to 2 weeks post RT): 49 % Subacute: 73 % Late (after 6 months): 29 %	Median 2‑year 4‑year	86 months 91 % n/a	33 months 57 % n/a	n/a n/a 70 %
Weykamp et al., 2020 [20]	46 (58 met)	21	Retrospective, single arm	1: 80.4 % 2: 17.4 % 3: 1.7 % (> 3 met in total, but 1 progressive oligoprogression: 30 %)	Metachronous OMD 70 % Oligoprogression 30 %	Bone only 56.3 % Liver 25 % Brain 6.3 % More than 1 site 12.6 %	71.7 % (58.7 % endocrine)	1–10 fx/5–30 Gy per fraction	Grade 1: 16 % Grade 2: 2 % No grade ≥ 3	1‑year 2‑year	85.4 % 62.1 %	54.3 % 17 %	92.2 % 89 %
Onal et al. 2018 [32]	22 (26 met)	**–**	Retrospective, single arm	1: 86.4 % 2: 9.1 % 3: 4.5 %	Metachronous OMD 14 % Oligoprogression 86 %	Liver 100 % (31.8 % liver-only)	100 % chemotherapy (4 neoadjuvant, 18 postop)	3 fx/54 Gy	Grade 3: 9.1 % (1 rib fracture, 1 duodenal ulcer) No grade 4	Median 1‑year 2‑year	n/a 85 % 57 %	7.4 moths 38 % 8 %	n/a 100 % 88 %

*OMD* oligometastatic disease, *OS* overall survival, *PFS* progression-free survival, *LC* local control, *RT* radiotherapy, *fx* fractions, *met* metastases

Analyses from seven studies [10, 17–22] encompassing a sample size of 15–120 patients with 1–5 metastases (mostly ≤ 2 lesions in 70.9–100 % of cases) reported LC rates at 1 and 2 years between 86.9 and 100 % and 79.2 and 100 %, respectively. The 1‑ and 2‑year PFS ranged from 38 to 80 % (seven studies) and 8 to 75 % (nine studies), respectively. Further details on the results are shown in Table 1.

Eight studies provided graded toxicity data. No grade 3 toxicities were seen in four of eight studies. Grade 3 toxicities were seen in four studies (4.2–18 %).

A recent systematic review and meta-analysis summarizes most of these data in a quantitative manner [23].

### Outcome of patients with OMD from different primary tumors treated with SBRT

Overall, the abovementioned studies consistently show that local ablative radiotherapy in oligometastatic breast cancer patients leads to very high local control rates in the range of 90 % and, depending on disease- or patient-specific factors, more or less favorable PFS and OS. Randomized controlled trials of SBRT in patients with OMD demonstrated improvements in PFS and partly also in OS, especially in prognostically more favorable constellations [24–30]. However, these studies were not focused on breast cancer, but usually included different histologies. The SABR-COMET randomized phase II trial was the only trial to enroll a minority of breast cancer patients but did not report the results of the breast cancer subgroup separately [29, 30]. There was an imbalance in baseline characteristics, with more patients with prostate cancer randomized to the experimental arm. However, a post-hoc sensitivity analysis excluding patients with prostate cancer confirmed the findings in the overall cohort. The other randomized controlled trials were conducted in patients with lung or prostate cancer. While trials enrolling patients with prostate cancer mostly focused on metachronous oligorecurrence, lung cancer trials mainly enrolled patients with synchronous OMD. The interpretation of the data is not trivial due to the heterogeneity in tumor entities as well as previous and subsequent systemic therapy. Furthermore, almost all of the trials involving breast cancer patients were cohort studies without a control arm, rendering causal inferences impossible. In summary, there is evidence that SBRT is associated with high local control rates and low toxicity in patients with oligometastatic breast cancer. However, there is only indirect evidence that SBRT in OMD breast cancer may prolong survival in breast cancer patients. Further research is needed to define which breast cancer patients with OMD have a clinically relevant benefit from SBRT beyond local tumor control.

### Prognostic factors in patients with oligometastatic breast cancer

#### Bone versus no bone metastases

It is well documented that in breast cancer, bone-only metastases are associated with a better prognosis for LC, OS, and PFS than visceral or brain metastases, irrespective of treatment [10, 31, 32]. The 5‑ and 10-year OS after SBRT for patients with bone-only metastases compared to patients with metastatic lesions at other sites were 83 and 75 %, and 31 and 17 %, respectively [10]. Milano et al. furthermore demonstrated an LC rate after SBRT of bone metastases after 10 years of 100 % vs. 73 % for other metastatic sites [10]. For patients with bone-only metastases, David et al. reported a 2-year LC of 100 % [17]. In a study by Onal et al. including only patients with liver metastases with or without bone or lung metastases, the 2‑year LC, PFS, and OS were 88, 8, and 57 %, respectively [33]. In an analysis by Wijtunga et al. including patients with oligometastatic breast cancer treated with SBRT, the presence of visceral or brain metastases was associated with an inferior prognosis as compared to bone-only disease (HRs for PFS and OS of 2.19 and 1.77, respectively) [32]. Furthermore, the study by Scorsetti et al., including only patients with visceral metastases, suggests inferior PFS and OS as compared to studies of patients with bone-only OMD, despite good local control with SBRT [18].

#### Number of metastases

In general, the available data show that prognosis on survival endpoints declines with an increasing number of metastases. Regarding the prognostic influence of the number of metastases, Weykamp et al. identified a solitary metastasis as an independent prognostic factor for PFS (HR 0.363) and distant control (HR 0.186) compared to > 1 metastases [21]. Further, Steenbruggen et al., based on the Netherlands Cancer Registry (*n* = 3447) including 612 breast cancer patients with OMD, showed that patients with a maximum of 3 metastases vs. > 3 metastases had an estimated 10-year survival of 14.9 vs. 3.4 % [34]. The HRs for OS for patients with 1 metastasis, 2–3 metastases, and 4–5 metastases versus > 5 metastases were 0.70 (*p* = 0.03), 0.63 (*p* = 0.009), and 0.91 (n. s.), respectively. In this analysis, however, only 20 % of patients received local ablative treatment to metastatic sites, which mostly consisted of SBRT. Again, it should be emphasized that patients with 3–5 metastatic lesions were underrepresented in most studies. In the publications mentioned in Table 1, 70–100 % of the patients had a maximum of 2 metastases.

#### Time of metastases

Whether the timing of OMD diagnosis plays a prognostic role is still a matter of debate in breast cancer. Dawood et al. analyzed a large cohort of metastatic breast cancer patients, albeit without mentioning the number of OMD patients. The results suggest that patients with synchronous metastases have a significantly better OS than those with metachronous metastases [31]. Interestingly, if the metastases occurred > 5 years after the initial diagnosis, the difference in survival was not significantly different compared to patients with synchronous metastases. Also, Wijtunga et al. saw better OS rates for patients developing metastases > 5 years after primary diagnosis vs. < 5 years (HR 4.17; *p* = 0.003) in the OMD setting [32].

In this context, systemic therapy, often given initially as part of an adjuvant treatment regimen, plays an important role. Adjuvant therapy may select particularly aggressive or resistant cell clones, which then leads to an adverse prognosis of metachronous metastatic disease [35, 36]. In turn, a long disease-free interval from curative treatment until the development of metastatic disease seems to be prognostically favorable [31].

#### Further prognostic factors

Numerous further clinical factors are well known to influence the prognosis of breast cancer patients. Beside others, these include age, hormonal receptor status, HER2 status, clinical tumor stage at diagnosis, tumor grading, and the performance status of the patient [37]. The prognostic relevance of these factors has been established mainly in the context of studies dealing with systemic therapy. However, the extent to which these factors are also meaningful for SBRT of OMD is still far less clear. Kucharczyk et al. performed a systematic review exploring factors defining an OMD population that may benefit from ablative therapies including mostly surgery and SBRT [38]. Overall, 41 retrospective studies with 1813 breast cancer patients were identified. Of these patients, 113 had extracranial OMD, including 79 treated with ablative dose radiotherapy. No quantitative meta-analysis was conducted due to the heterogeneity of the included studies. Positive prognostic factors included tumor biology (hormone receptor positive/HER2 negative vs. other subtypes), longer disease-free interval before local ablative treatment, and number of lesions. The authors assessed that the existing evidence does not show a clear direction as to which patients can benefit from such a treatment approach. Further, the authors concluded that the use of an ablative therapy per se (versus no ablative therapy) is supported by low-level evidence.

This observation was confirmed in the previously mentioned database analysis by Steenbruggen et al., independent of systemic treatment [34]. Multivariate analyses revealed a survival benefit in favor of local ablative treatment (mostly SBRT) with an HR of 0.57 (*p* = 0.02) in patients with 1–3 breast cancer metastases. However, in the absence of randomization, a relevant bias in favor of patients receiving local ablative therapies cannot be excluded in this analysis.

### Tumor biology and systemic treatment

The benefit of local ablative therapy for survival is expected to be dependent on the propensity for further systemic progression and efficacy of systemic therapy. Not surprisingly, most studies have focused on patients with hormone receptor-positive and HER2-negative breast cancer, while especially patients with triple-negative breast cancer (TNBC) were underrepresented. Just recently, Wijetunga et al. highlighted the association between clinical outcomes and clinical and molecular factors in a cohort treated with SBRT [32]. Overall, 79 breast cancer patients with 103 metastatic lesions were identified. Patients with HR+/HER-, HER2+ (irrespective of HR expression), and TNBC breast cancer had a median OS of 86 months, not reached, and 18 months, respectively. Tan et al. confirmed the poor prognosis of patients with TNBC treated with SBRT [22].

Response to systemic therapy may be another important factor to consider. There has been growing interest in the use of local ablative treatment in patients with oligoprogression during systemic therapy. Mainly, this has been used as a means to keep patients on the same systemic therapy regimen while treating progressing metastases locally. Contrary to the concept of treating all existing metastatic lesions with local ablative therapy, these treatments are often applied to progressive lesions only. It is important to mention that the term “oligoprogression” may include patients with OMD as well as patients with polymetastatic disease. The abovementioned ESTRO-EORTC classification of OMD specifically designates the term “induced OMD” for patients with prior history of polymetastatic disease [12].

Oligoprogressive disease has a comparatively worse prognosis. Tan et al. demonstrated that for patients treated with SBRT, those with oligoprogressive disease had an inferior prognosis as compared to patients with OMD, with 1‑ and 2‑year PFS rates of 19.6 and 8 % compared to 66 and 52 %, respectively [22]. Despite this, or even because of it, oligoprogression may be a particularly interesting subclass for local therapies within OMD. For example, Tan et al. pointed out that SBRT as an “alternate line of therapy” may delay the potential unfavorable effects on quality of life with regard to toxicities due to switching systemic therapy [22].

Preliminary data from a randomized phase II trial, the CURB trial, were presented by Tsai et al. [16]. Latest interim results presented by Tsai et al. at the ASTRO Annual Meeting 2021 must be considered with caution due to inclusion of different entities—NSCLC and breast cancer—with different outcomes of the small subgroups. The data favored the addition of SBRT to oligoprogressive lesions but could not demonstrate a benefit from additional SBRT in the subgroup of patients with oligoprogressive breast cancer. However, it should be emphasized that only 44 breast cancer patients have been analyzed so far (22 patients per arm), including 16 patients with TNBC. Further, it should be underscored here that patients included in the study by Tsai et al. were at a high risk per se on the one hand, and still oligoprogressive on the other. This is probably an unfavorable subgroup of patients for SBRT. A general recommendation in one direction or the other cannot be derived from these preliminary data of a very specific high-risk population. However, this demonstrates once again the importance of developing meaningful selection criteria for SBRT in OMD as well as appropriate subclassifications as proposed by Guckenberger et al. [12].

In general, systemic therapy is generally recommended in the setting of metastatic breast cancer. As expected, systemic therapies were applied before and also after SBRT in the vast majority of cases in the considered studies (Table 1). It is currently unclear whether there may be patients with a favorable prognosis and a low propensity for further systemic spread (i.e., metachronous oligorecurrence with a long treatment-free interval), where SBRT may be used as the sole treatment. In addition, it is not clear whether SBRT to oligoprogressive sites may allow for continuation of systemic therapy beyond progression. Here, however, it should be mentioned again that local ablative therapy of the metastases per se was recently shown by Steenbruggen et al. to be a favorable prognostic factor, independent of the systemic treatment [34]. However, when interpreting the retrospective observational study of Steenbruggen et al., a possible bias must be taken into account.

When combining SBRT and concomitant systemic therapy, possible interactions in terms of toxicity need to be considered. There is a lack of prospective clinical trials in this regard. A systematic review published in 2017 provides an overview of toxicity data of SBRT in combination with targeted agents and immunotherapy [39]. There were signs of potentially increased toxicity of SBRT in combination with EGFR-targeting tyrosine kinase inhibitors and bevacizumab. For CDK4/6 inhibitors there is conflicting evidence, with some reports of increased toxicity; however, these mostly included patients treated with standard treatment techniques and not SBRT [40].

There has been growing interest in the combination of radiotherapy and immunotherapy, also in breast cancer [41]. Currently, the use of immunotherapy in breast cancer outside of clinical trials is limited to patients with TNBC. A recent analysis conducted by the Food and Drug Administration did not show major increases in toxicity in patients treated with radiotherapy followed by immunotherapy when compared to immunotherapy alone based on data of more than 15000 patients of which approximately 3000 patients received radiotherapy [42].

A patterns-of-care survey among German-speaking countries established by the German Society for Radiation Oncology (DEGRO) working group for radiosurgery and stereotactic radiotherapy assessed patterns of care regarding combinations of systemic therapy and SBRT [43]. The majority of clinics pause targeted therapy or immunotherapy 1 week before and after SRT, irrespective of the type of systemic therapy given. However, a recent retrospective analysis of 158 patients by the same group did not show a difference in toxicity according to whether systemic therapy was interrupted during radiotherapy or not [44]. Overall, the rate of acute and chronic grade 3+ toxicity was below 5 %.

In clinical practice, an individual assessment based on treatment site, expected dose to organs at risk, type of systemic therapy, and risk of systemic disease progression is necessary. A multidisciplinary discussion is advised. Prolonged discontinuation of systemic therapy should be avoided wherever possible.

### Primary tumor control

Older retrospective data suggest that there is a survival benefit for local treatment including surgery in stage IV breast cancer [45]. However, recent prospective randomized studies have shown that in the case of synchronous metastasis, radical therapy of the primary breast tumor does not provide a prognostic advantage [46–48]. However, these trials were not specifically conducted in patients with OMD. Furthermore, these trials did not include local treatment to metastatic sites.

In relevant studies, a controlled primary tumor is a prerequisite for SBRT of OMD in breast cancer [20, 24, 31]. In addition, only patients with controlled primary tumors were eligible for the randomized SABR-COMET study [29]. Generally, in a concept in which all pathological lesions are treated ablatively, it seems logical to also treat the primary ablatively.

### Current clinical trials

An overview of randomized controlled trials enrolling only patients with breast cancer is given in Table 2. Radiotherapy is the predominant local treatment modality. Trials include patients with 3–5 metastases; however, three trials have additional limits including maximum size and/or volume of the metastatic lesions. Three out of five trials are limited to the first-line metastatic setting while only one trial restricts enrollment in terms of tumor biology. Progression-free and overall survival are the most common primary endpoints, although quality of life is used as a co-primary endpoint in the OLIGOMA trial [49].

**Table 2:** Randomized controlled trials of local treatment in patients with oligometastatic breast cancer

–	OLIGOMA (NCT04495309)	NRG-BR002 (NCT02364557)	STEREO-SEIN (NCT02089100)	OMIT (NCT04413409)	Chinese Academy of Sciences (NCT04646564)	LARA NCT04698252
Sample size	564 patients	402 patients (phase II/III)	280 patients	172 patients	170 patients	74 patients
Maximum number of metastatic lesions	5	4	5 (≤ 10 cm/≤ 50 ml)	3 (only lung or liver metastases, < 5 cm)	5 (≤ 5 cm)	4 (bone/lung/liver), ipsilateral cervical or contralateral axillary metastases
Setting	Any treatment line	First-line setting, maximum of 1 year after diagnosis of MBC	First-line metastatic setting, HR positive	First-line setting	Metachronous recurrence > 3 months after surgery	Stable disease after 6 months of systemic therapy; HR positive, HER2 negative
Type of local therapy	Radiotherapy	Radiotherapy, surgery	Radiotherapy	Surgery	Radiotherapy	Radiotherapy, surgery, radiofrequency ablation
Primary endpoint	PFS + QoL	PFS/OS	PFS	OS	PFS	PFS

*HR* hormone receptor, *PFS* progression-free survival, *OS* overall survival, *QoL* quality of life

Interestingly, there are also few trials on oligoprogressive breast cancer. The AVATAR trial (NCT04530513) is a phase II trial of patients with up to five oligoprogressive sites during treatment with endocrine therapy and a CDK4/6 inhibitor [50]. All patients will receive local ablative radiotherapy to the progressing lesions. The time to change of systemic therapy, measured from the commencement of SBRT to change in systemic therapy, was selected as the primary endpoint. Repeat local ablative radiotherapy is allowed in the case of new oligoprogressive lesions.

Details regarding radiotherapy treatment regimens are only available for NRG BR-002, AVATAR, and OLIGOMA. While SBRT in 1–5 fractions is mandated in NRG BR-002 and AVATAR, there is some flexibility to use more protracted regimens under specific clinical circumstances (e.g., large lesions, locoregional recurrences, spinal cord compression) in the OLIGOMA trial, in part due to the broader inclusion criteria [49]. Nevertheless, SBRT is strongly favored in the OLIGOMA trial.

### Recommendations

Based on the discussed data, recommendations of the DEGRO breast cancer expert panel as well as the level of evidence depending on the classification/subclassification of OMD and clinical presentation are given in Table 3. An interdisciplinary tumor board should be involved.

**Table 3:** Recommendations for stereotactic body radiation therapy (SBRT) in breast cancer patients with extracranial oligometastatic disease (OMD)

Classification of OMD	Subclassification of OMD	Subclassification of OMD	Subclassification of OMD	Clinical presentation	DEGRO recommendation for SBRT^b^	Level of evidence
**Genuine oligometastatic disease**	**De novo oligometastatic disease**	*Synchronous oligometastatic disease*		*1 lesion, bone* *1 lesion, elsewhere* *2–5 lesions*	++ + ±^a^	*4* *4* *4*
		*Metachronous oligometastatic disease*	Metachronous oligorecurrence	1 lesion, bone 1 lesion, elsewhere 2–5 lesions	++ + ±^a^	4 4 4
			Metachronous oligoprogression	1 lesion, bone 1 lesion, elsewhere 2–5 lesions	++ + ±^a^	4 4 4
	**Repeat oligometastatic disease**	*Repeat oligorecurrence*		*1 lesion, bone* *1 lesion, elsewhere* *2–5 lesions*	*No recommendation*	*Insufficient evidence*
		*Repeat oligometastatic disease*	Repeat oligopersistence	1 lesion, bone 1 lesion, elsewhere 2–5 lesions	No recommendation	Insufficient evidence
			Repeat oligoprogression	1 lesion, bone 1 lesion, elsewhere 2–5 lesions	±^a^ ±^a^ ±^a^	5 5 5
**Induced oligometastatic disease**		*Induced oligorecurrence*		*1 lesion, bone* *1 lesion, elsewhere* *2–5 lesions*	*No recommendation*	*Insufficient evidence*
		*Induced oligometastatic disease*	Induced oligopersistence	1 lesion, bone 1 lesion, elsewhere 2–5 lesions	No recommendation	Insufficient evidence
			Induced oligoprogression	1 lesion, bone 1 lesion, elsewhere 2–5 lesions	±^a^ ±^a^ ±^a^	4 4 4

For definition of oligometastatic disease (*OMD*), see Guckenberger et al. [12].

^a^better results in patients with long progression-free interval, no visceral lesions, or ≤ 3 lesions

^b^if all lesions are amenable for local ablative stereotactic body radiation therapy (SBRT)

++ SBRT should be performed, + SBRT should be considered, ± SBRT may be considered in favorable cases (see^a^)

Adequate classification of OMD [12] and judicious patient selection, also considering systemic therapy, are of high importance in this context.

Essential requirements for ablative radiotherapy are the implementation of adequate treatment planning, dosage, and dose prescription as well as the highest standard of technical quality, quality assurance, and documentation. Regarding these requirements, we refer to the recommendations and guidelines published by the DEGRO/DGMP [51, 52]. Careful consideration of organ dose constraints from consensus publications and international collaborations is strongly advised [53–56].

## Conclusion

Local ablative radiotherapy is a promising treatment option for patients with oligometastatic breast cancer, offering high rates of local control with very limited toxicity. Currently, prospective cohort studies show very favorable results in selected patients. Randomized controlled trials are needed to demonstrate an additional benefit of SBRT in the setting of optimal systemic therapy. The use of SBRT in patients with oligometastatic breast cancer should always be discussed in a multidisciplinary conference.
